# Mass spectral proteomics checklist

**DOI:** 10.1016/j.jbc.2026.111286

**Published:** 2026-03-24

**Authors:** Robert S. Haltiwanger, Roger J. Colbran, Henrik Dohlman, Lee Graves, Lance Wells

The *Journal of Biological Chemistry* (*JBC*), like all ASBMB journals, is committed to publishing papers that maintain the highest standards of Rigor, Reproducibility, and Transparency (https://www.jbc.org/article/S0021-9258(17)48765-5/fulltext) in data reporting. Over the last few years, we have had a significant increase in the number of manuscript submissions that incorporate mass spectral proteomics data as just one part of multidisciplinary papers. Our long-standing guidelines for such studies have been that the data should be reported according to the standards used at our ASBMB sister journal, *Molecular & Cellular Proteomics* (*MCP*). *MCP* standards were maintained using an extensive and rigorous checklist that was developed by and for experts in the field. Completion of the *MCP* checklist was a daunting proposition to the authors of many related *JBC* manuscripts, who are not necessarily card-carrying experts in the use of these methods and reporting of the data generated. Accordingly, our working group of Associate Editors and Editorial Board Members with related expertise was charged with the development of a more author-friendly, streamlined *JBC* checklist that retained the essential elements of the *MCP* checklist, and emphasized the crucial parameters for reporting mass spectral proteomics data.

The new checklist was recently activated. *JBC* now requires submission of the completed checklist with manuscripts containing mass spectral proteomics data when the first revision is submitted. The purpose of the checklist is to remind authors to include in their manuscript complete descriptions of the methods (for Rigor and Reproducibility) and the raw data (Transparency). The checklist can be accessed here (https://legacyfileshare.elsevier.com/promis_misc/jbc-proteomics-checklist.pdf). It includes sections on Source and Isolation of Biological Samples, Mass Spectrometry Conditions, Data Analysis and Processing Methods, Statistics and Data Validation, Data Reporting, and Predictions from Proteomics Data and Presentation. These methods will allow other scientists to replicate your data, and the raw data will allow other scientists to confirm your interpretations of the data and perform additional searches/analyses that were not reported in the manuscript.

## Conflict of interest

The authors declare that they have no conflicts of interest with the contents of this article.

